# Effects of tillage management on soil organic carbon mineralization under double cropping rice system of southern China

**DOI:** 10.1038/s41598-024-72042-6

**Published:** 2024-09-10

**Authors:** Haiming Tang, Lihong Shi, Li Wen, Kaikai Cheng, Chao Li, Weiyan Li, Xiaoping Xiao

**Affiliations:** Hunan Soil and Fertilizer Institute, Changsha, 410125 People’s Republic of China

**Keywords:** Conventional tillage, Crop residue, Paddy field, SOC mineralization, Soil enzyme, Microbiology, Environmental sciences

## Abstract

Soil organic carbon (SOC) plays a vital role in maintaining or enhancing soil fertility and quality of paddy field, but there is still limited information about how SOC mineralization responds to different tillage managements under the double-cropping rice (*Oryza sativa* L.) system in southern of China. Therefore, this study was designed to explore the changes in SOC content, soil enzyme activities (invertase, cellulose and urease), SOC mineralization at 0–10 cm and 10–20 cm layers and its relationship with 7-years tillage management under the double-cropping rice system of southern China. The experiment included four tillage managements: rotary tillage with all residues removed as a control (RTO), conventional tillage with residue incorporation (CT), rotary tillage with residue incorporation (RT), and no-tillage with residue retention (NT). The results indicated that SOC and soil labile organic carbon contents at 0–10 cm and 10–20 cm layers in paddy field with CT and RT treatments were significantly higher than the RTO treatment. Compared to the RTO treatment, SOC mineralization and accumulation at 0–10 cm and 10–20 cm layers in paddy field with CT, RT and NT treatments were increased. SOC accumulation and potential mineralization at 0–10 cm layer with NT treatment were significantly higher than the CT, RT and RTO treatments. Soil mineralization constant at 10–20 cm layer with CT treatment was significantly higher than those of RT, NT and RTO treatments. This result indicated SOC mineralization rate and accumulation at 10–20 cm layer of CT, RT, NT and RTO treatments were lower than those of treatments at 0–10 cm layer. Compared to RTO treatment, soil invertase, cellulose and urease activities with CT and RT treatments were significantly increased. Compared to RTO treatment, soil invertase, cellulose and urease activities at 0–20 cm layer of CT treatment increased by 22.6%, 46.2% and 89.0%, respectively. There was significantly positive correlation between SOC accumulation and SOC content, soil invertase, cellulose, urease activities, but SOC accumulation was significantly negative correlated with soil pH, bulk density. Therefore, CT and RT treatments were beneficial managements to improve SOC content and SOC mineralization in the double-cropping rice field of southern China.

## Introduction

Soil organic carbon (SOC) plays a key role in keeping or enhancing quality and fertility of agricultural soil^1^. It general accepted that SOC change is regarded as a vital indicator in evaluating soil quality because SOC is tight associated with biogeochemical cycle of major soil nutrients and provided energy and substrate for soil microbial activities^2^. Soil labile organic carbon (LOC) content (eg., light fraction organic carbon (C)) is also a vital component of organic C that is easy to decompose by soil microorganism, and is closely related with soil nutrients and is a vital indicator in changing of soil property^3^. Higher SOC content improves soil quality and represents substantial contribution via C sequestration that reduces carbon dioxide (CO_2_) emission^4^. Therefore, it was beneficial managements to improve soil quality and reduce greenhouse gases emission from agricultural soil by increased of SOC content.

In the previous studies, these results proved that there was close correlation between SOC mineralization and field managements, including tillage, fertilizer regime, crop residue, cropping system^5,6^. It general accepted that SOC mineralization was decreased by enhancing SOC sequestration and reducing CO_2_ emission to atmosphere^7^. SOC accumulation was regulated by balance between C input from crop residue and C output from SOC mineralization^8^. It is demonstrated that soil structure, physiochemical and biological characteristics, SOC mineralization were changed under different tillage conditions^6,9,10^. It is found that conservation tillage (CT) can enhance SOC accumulation at soil surface^11^. The result indicated that no-tillage (NT) decrease SOC mineralization compared with plow tillage (PT) treatment^12^. However, other result found that SOC mineralization rate with NT treatment was increased^13^. Contrary, Liu et al.^14^ found there was not obvious difference in SOC mineralization between NT and CT treatments. These contradictory results were associated with local field environmental conditions^6^. Therefore, there is still needed to investigate SOC mineralization, accumulation, and responses to soil chemical properties with different tillage managements.

Rice (*Oryza sativa* L.) is the main crop in tropical and subtropical monsoon climate regions of Asia^15^. Double-cropping rice system is including early rice and late rice in a year, and this rice system is main cropping system in southern of China, and tillage with crop residue incorporation managements is an important factor in influencing on soil quality and fertility of paddy field. In order to explore the effects of different tillage managements on soil physicochemical properties in paddy field, a tillage experiment were set up in the double-cropping rice field of southern China, and the experiment included four tillage treatments: conventional tillage with crop residue incorporation (CT), rotary tillage with crop residue incorporation (RT), no-tillage with crop residue retention (NT), and rotary tillage with all crop residues removed as a control (RTO). In our previous study, this result found that soil chemical properties (eg., soil pH, SOC content and SOC stock) at plow layer (0–20 cm) with CT, RT and NT treatments were increased, compared to RTO treatment^16^. However, it is still need to further investigate SOC mineralization and accumulation at 0–10 cm and 10–20 cm layers responds to different tillage managements, correlation relationship between SOC mineralization and soil physicochemical properties, enzyme activities under the double-cropping rice system in southern of China. We hypothesize that soil LOC, SOC mineralization and accumulation at 0–10 cm and 10–20 cm layers in the double-cropping rice field with CT, RT and NT treatments were increased, compared to RTO treatment. Therefore, the objectives of this study were: (i) to explore the effects of different tillage managements on SOC mineralization and accumulation at 0–10 cm and 10–20 cm layers in the double-cropping rice field, (ii) to analyze the relationship between SOC mineralization and soil enzyme activities (invertase, cellulose and urease activities), physicochemical properties (soil bulk density, soil pH, SOC, NH_4_^+^–N and NO_3_^−^–N contents) under the double-cropping rice system of southern China.

## Materials and methods

### Sites and cropping system

The field experiment was begun in November 2015. It was located in Ningxiang City (28°07′ N, 112°18′ E) of Hunan Province, China. The field experiment was under continental monsoon climate, monthly mean temperature was 17.2 °C, annual mean precipitation and evapotranspiration were 1553 and 1354 mm, respectively. At the beginning of this field experiment, soil characteristics at 0–20 cm layer were showed as following: soil organic carbon (SOC) 22.1 g kg^−1^, total nitrogen (N) 2.1 g kg^−1^, available N 192.2 mg kg^−1^, total phosphorous (P) 0.8 g kg^−1^, available P 13.5 mg kg^−1^, total potassium (K) 13.2 g kg^−1^, and available K 81.9 mg kg^−1^, pH 5.8. The other more detail information about soil type and soil texture at 0–20 cm layer in paddy field, and cropping system were described as by Tang et al. (2019)^16^.

### Experimental design

The field experiment included four tillage treatments: conventional tillage with crop residue incorporation (CT), rotary tillage with crop residue incorporation (RT), no-tillage with crop residue retention (NT), and rotary tillage with all crop residues removed as a control (RTO). The area of each plot was 56.0 m^2^ (7 m × 8 m), and each tillage treatment was laid out in a randomized complete block design with three replications. For the RT and RTO treatments, a 20–25 cm deep chisel plough with a 20 cm distance between the tines was used once following the application of tillage^17^. Chinese milk vetch and rice straw residue were retained for the CT, RT and NT treatments when both the Chinese milk vetch, early and late rice crop residue were retuning to paddy field. The number of Chinese milk vetch, early and late rice crop residue retuning to paddy soil for the CT, RT and NT treatments were 22,500, 2000, and 2000 kg hm^−2^, respectively. Chinese milk vetch and rice crop residue were cutted into 5–10 cm by residue cutting machine and incorporated into the soil with tillage management under CT and RT treatments. CT treatment was tilled once with moldboard plow at depth of 15–20 cm and then rotovated twice at depth of 8–10 cm before transplanting of rice seedling. RT and RTO treatments were rotovated four times at the depth of 8–10 cm before transplanting of rice seedling. It were ensure that our have permission to the collect rice seedlings. RTO treatment was similar with those of RT treatment except that all crop residues were removed from paddy field both early rice and late rice whole growing season.

The total quantity of N were applied at the rate of 150 and 180 kg hm^−2^ (60% and 40% at basal and tillering stages), 75 kg hm^−2^ of P_2_O_5_ as superphosphate, and 120 kg hm^−2^ of K_2_O as potassium chloride during early rice and late rice whole growing season, respectively. All the P_2_O_5_ and K_2_O fertilizer were applied at tillage before rice transplanting. The kinds of fertilizer were urea, ordinary superphosphate and potassium chloride, respectively. The other more detail information about application of the crop residue and chemical fertilizer, rice varieties, rice transplant and harvest were described as by Tang et al.^16^.

### Soil sampling

Soil samples were collected at the maturity stage of late rice in October 2021. Soil samples close to the rice plant were collected at 0–10 cm and 10–20 cm layers in paddy field. Correspondingly, one composite soil sample was collected from ten points with soil sampler (diameter was 4 cm) with each plot. Thus, three soil composite samples were collected for each tillage treatment. Then the mixed soil samples were air dried at room temperature (12 d) and sieved through a 2 mm mesh for chemical analysis in laboratory.

### Soil laboratory analysis

#### Soil physicochemical properties

SOC content was determined using the rapid titration method (wet combustion method) as described by Ellert and Bettany^18^. Soil labile organic carbon (light fraction organic C (LOC)) content was determined using the density fractionation method described as by Janzen et al.^19^. Soil pH was determined electrometrically in a suspension with 1:2 (g ml^−1^) soil–water relationships at 25 °C. Soil bulk density (ρb) was determined using metallic core of known volume (having 15 cm internal diameter and 20 cm length) method introduced as by Blake and Hartge^20^.

Soil enzyme activities (invertase, cellulose and urease activities) of the soil sample were investigated according to the method introduced as by Zhang et al.^21^. Soil NH_4_^+^–N and NO_3_^−^–N contents of the soil sample were followed by flow injection analysis^22^.

#### Soil carbon mineralization

Soil carbon mineralization was investigated in an aerobic incubation experiment. Briefly, 10.0 g soil sample (< 2 mm) was fully placed in 100 ml incubation vessel with deionized water (80 ml). Without any soil sample input was regarded as blank. During incubation period, incubation of all soil samples were carried out in a dark, temperature controlled incubator at 28 °C conditions. After 1, 2, 3, 6, 10, 14 d and then weekly to 200 d, carbon dioxide (CO_2_) of all soil samples were measured by titrating the NaOH solution with 0.1 M HCl in BaCl_2_.

Assuming no priming effect in the control soil, the quantity of C evolved as CO_2_ from the soil samples were calculated by subtracting the amount produced in the control soil from that produced with deionized water treated soil and expressed as a percentage of the total organic carbon (TOC) concentration of the soil samples^23^. Then the SOC mineralization rate and accumulation of all soil samples were calculated based on the production of CO_2_.

The accumulation and mineralization process of SOC with different tillage treatments were fitted with following equation:

$$ y = \frac{{{\text{cp}}}}{{1 - {\text{exp}}\left( { - {\text{kt}}} \right)}} $$

where, *y* was accumulation of SOC (mg kg^−1^); Cp was potential mineralization of SOC (mg kg^−1^); k was mineralization constant (d^−1^), t was day of mineralization (d).

All the methods in laboratory were carried out in accordance with relevant Institutional guidelines.

### Statistical analysis

The investigate items of each tillage treatment mean were compared by using one-way analysis of variance (Anova) following standard procedure at 5% probability level. The least significantly difference (LSD) test was used to identify significantly differences among the tillage treatments mean in terms of each investigate items. The statistical analysis of data in this manuscript was conducted by using SAS 9.3 software package^24^. The results of all investigate items were expressed as mean and standard error, and with three replications. In this manuscript, different tillage treatments (CT, RT, NT and RTO) were regarded as fixed variable, crop residue incorporation and with all crop residues removed treatments were regarded as random variable. The relationships between SOC mineralization accumulation and soil enzyme activities, soil physicochemical properties were analyzed by using Pearson’s correlation.

## Results

### SOC and labile organic carbon contents

**Fig. 1:**
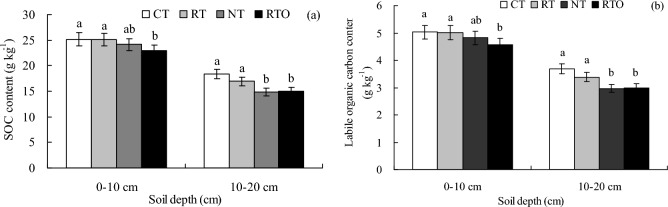
Effects of different tillage treatments on soil organic carbon (**a**) and labile organic carbon content (**b**) in a double-cropping rice field. CT: conventional tillage with crop residue incorporation; RT: rotary tillage with crop residue incorporation; NT: no-tillage with crop residue retention; RTO: rotary tillage with all crop residues removed as a control. SOC: soil organic carbon. Different lowercase letters indicated significantly differences (*p* < 0.05) among different tillage treatments. The same as below.

This result indicated that SOC contents at 0–10 cm and 10–20 cm layers in the double-cropping rice field were obvious affected by the different tillage treatments (Fig. 1a). Compared with RTO treatment, SOC content at 0–10 cm layer with CT and RT treatments were significantly increased (*p* < 0.05), SOC content with CT and RT treatments increased by 9.9% and 9.6%, respectively. This result proved that SOC contents at 10–20 cm layer with CT and RT treatments were larger than that of NT and RTO treatments, but there was no significantly (*p* > 0.05) difference in SOC content at 10–20 cm layer in paddy field between NT and RTO treatments. This result proved that SOC contents at 10–20 cm layer with CT, RT, NT and RTO treatments were lower than that of 0–10 cm layer.

Soil LOC contents at 0–10 cm and 10–20 cm layers in paddy field with all tillage treatments were between 3.00 and 5.03 g kg^−1^ (Fig. 1b). At soil 0–10 cm layer, LOC content with RTO treatment were significantly lower (*p* < 0.05) than that of CT and RT treatments. Compared with RTO treatment, LOC content with CT and RT treatments increased by 9.9% and 9.4%, respectively. At soil 10–20 cm layer, LOC contents with NT and RTO treatments were significantly lower (*p* < 0.05) than that of CT and RT treatments. This result proved that LOC content at 10–20 cm layer with all tillage treatments were lower than that of 0–10 cm layer.

### Soil carbon mineralization rate

**Fig. 2:**
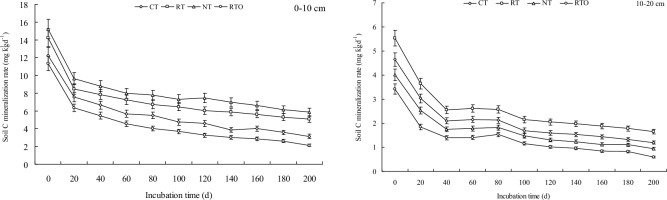
Effects of different tillage treatments on soil carbon mineralization rate in a double-cropping rice field.

At the initial stage of incubation, SOC mineralization rate was decreased rapidly and then tended to be flat (Fig. 2). This result indicated that SOC mineralization rates at 0–10 cm and 10–20 cm layers in paddy field with all tillage treatments were the highest at first day of incubation. At soil 0–10 cm layer, SOC mineralization rate with NT treatment was significantly higher (*p* < 0.05) than that of the other tillage treatments, SOC mineralization rate with NT treatment was 15.22 mg kg^−1^ d^−1^. The result showed that SOC mineralization rates at 10–20 cm layer with all tillage treatments were lower than that of 0–10 cm layer.

**Fig. 3:**
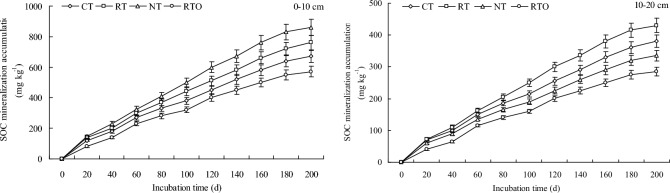
Effects of different tillage treatments on SOC mineralization accumulation in a double-cropping rice field.

SOC mineralization accumulation at 0–10 cm and 10–20 cm layers in paddy field with all tillage treatments were increased with the development of incubation time. The result showed that SOC mineralization accumulation at 10–20 cm layer with all tillage treatments were lower than that of 0–10 cm layer. At soil 0–10 cm and 10–20 cm layers, SOC mineralization accumulation with different tillage treatments were followed the order: NT > RT > CT > RTO, RT > CT > NT > RTO, respectively (Fig. 3).

### SOC mineralization characteristics

This result showed that soil potential mineralization (Cp) and mineralization constant at 0–10 cm and 10–20 cm layers in paddy field of CT, RT and NT treatments were significantly higher (*p* < 0.05) than that of RTO treatment (Table 1). Soil Cp at 0–10 cm layer in paddy field with NT treatment were significantly higher (*p* < 0.05) than that of CT, RT and RTO treatments. At 0–10 cm and 10–20 cm layers, mineralization constant of CT treatment were significantly higher (*p* < 0.05) than that of RT, NT and RTO treatments. Meanwhile, this result showed that ratio of potential mineralization/SOC at 0–10 cm and 10–20 cm layers in paddy field with CT, RT and NT treatments were significantly higher (*p* < 0.05) than that of RTO treatment. At soil 0–10 cm and 10–20 cm layers, ratio of potential mineralization/SOC with different tillage treatments were followed the order: NT > RT > CT > RTO, RT > CT > NT > RTO, respectively.

**Table 1:** Parameters of the first-order kinetics for SOC mineralization with different tillage treatments.

Soil layers (cm)	Treatments	SOC mineralization accumulation (mg kg^−1^)	Fitting parameter			Potential mineralization /SOC (%)
			Potential mineralization(mg kg^−1^)	Mineralization constant	R^2^	
0–10 cm	CT	640.13 ± 18.47c	1037.05 ± 29.93c	4.25 ± 0.12a	0.994	4.14
	RT	720.84 ± 20.81b	1352.62 ± 39.04b	3.17 ± 0.09b	0.986	5.37
	NT	830.35 ± 23.96a	1562.34 ± 45.10a	2.82 ± 0.08c	0.996	6.48
	RTO	550.67 ± 15.89d	875.62 ± 25.27d	2.35 ± 0.06d	0.992	3.83
10–20 cm	CT	360.47 ± 10.41b	350.64 ± 10.12b	6.29 ± 0.18a	0.987	1.90
	RT	415.15 ± 11.98a	520.83 ± 15.03a	4.53 ± 0.13c	0.993	3.08
	NT	320.05 ± 9.23c	246.57 ± 7.11c	5.36 ± 0.15b	0.986	1.66
	RTO	275.38 ± 7.94d	206.13 ± 5.95d	3.67 ± 0.11d	0.991	1.38

CT: conventional tillage with crop residue incorporation; RT: rotary tillage with crop residue incorporation; NT: no-tillage with crop residue returning; RTO: rotary tillage with all crop residues removed as a control. SOC: soil organic carbon.

Values were presented as mean ± standard error.

Different lowercase letters indicated significantly differences (*p* < 0.05) among different tillage treatments. The same as below.

### Soil enzyme activities and correlation with soil physicochemical properties

**Fig. 4:**
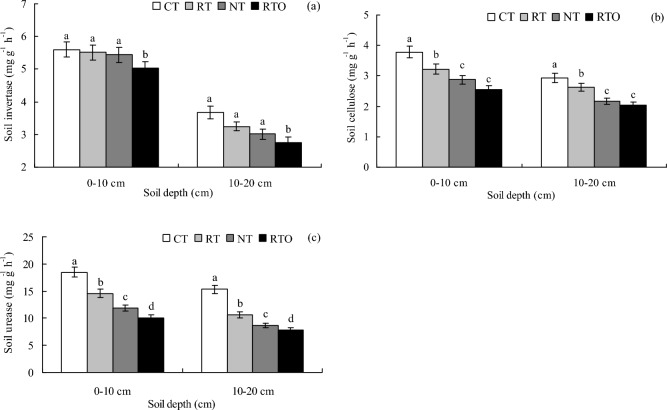
Effects of different tillage treatments on soil enzyme activities in a double-cropping rice field. (**a**) soil invertase activity; (**b**) soil cellulose activity; (**c**) soil urease activity.

This result proved that soil invertase activity at 0–10 cm and 10–20 cm layers in paddy field with CT, RT and NT treatments were significantly higher (*p* < 0.05) than that of RTO treatment, but there was no significantly difference (*p* > 0.05) in soil invertase activity between CT, RT and NT treatments (Fig. 4a). Soil cellulose and urease activities at 0–10 cm and 10–20 cm layers in paddy field with CT and RT treatments were significantly (*p* < 0.05) higher than that of RTO treatment (Fig. 4b,c). Compared to RTO treatment, soil cellulose activity 0–10 cm and 10–20 cm layers with CT treatment increased by 48.8% and 43.6%, respectively. This result demonstrated that soil urease activities at 0–10 cm and 10–20 cm layers with CT treatment were 1.83 and 1.95 times higher than that of RTO treatment, respectively.

There were significantly negative correlations between soil bulk density and SOC, NO_3_^−^–N contents, soil invertase, cellulose activities. There were significantly negative correlations between soil pH and SOC, NH_4_^+^–N, NO_3_^−^–N contents, soil invertase, cellulose, urease activities. There was significantly positive correlation between SOC content and NH_4_^+^–N, NO_3_^−^–N contents, soil invertase, cellulose, urease activities. There was significantly positive correlation between soil invertase activity and soil cellulose, urease activities (Table 2).

**Table 2:** Correlation relationship between soil enzyme activities and soil physicochemical properties with different tillage treatments.

Items	Bulk density	pH	SOC	NH_4_^+^–N	NO_3_^−^–N	Soil invertase	Soil cellulose
ρb	1						
pH	0.305	1					
SOC	− 0.602*	− 0.716*	1				
NH_4_^+^–N	0.154	− 0.642*	0.413*	1			
NO_3_^−^–N	− 0.415*	− 0.436*	0.404*	− 0.137	1		
Soil invertase	− 0.506*	− 0.775**	0.925**	0.463*	0.327	1	
Soil cellulose	− 0.473*	− 0.682**	0.874**	0.316	0.506**	0.813**	1
Soil urease	− 0.248	− 0.617**	0.839**	0.105	0.228	0.785**	0.567**

ρb: Soil bulk density; NH_4_^+^–N: ammonium nitrogen; NO_3_^−^–N: nitrate nitrogen.

* and ** were indicated significantly differences at 0.05 and 0.01 levels, respectively.

The same as below.

### Correlation relationship between SOC mineralization and soil physicochemical properties

This result indicated that SOC mineralization accumulation was significantly positively correlated with SOC content, soil invertase, cellulose, urease activities, but was significantly negatively correlated with soil pH, bulk density. Meanwhile, soil potential mineralization was significantly positively correlated with SOC content, soil invertase, cellulose, urease activities. However, this result showed that soil mineralization constant was significantly negatively correlated with SOC content, soil invertase, cellulose activities (Table 3).

**Table 3:** Correlation relationship between SOC mineralization accumulation, Cp, k and soil enzyme activities with different tillage treatments.

Items	pH	Bulk density	SOC	NH_4_^+^–N	NO_3_^−^–N	Soil invertase	Soil cellulose	Soil urease
*y*	− 0.728**	− 0.523*	0.926**	0.254	0.367	0.837**	0.811**	0.665**
Cp	− 0.703**	− 0.318	0.853**	0.362	0.230	0.806**	0.735**	0.614**
k	0.506*	0.472*	− 0.715**	− 0.376	− 0.339	− 0.638**	− 0.603**	− 0.306

*y*: SOC mineralization accumulation; Cp: potential mineralization; k: mineralization constant.

** were indicated significantly differences at 0.01 level.

## Discussions

### Effects of different tillage managements on SOC content

In the previous studies, those results indicated that soil organic carbon (SOC) and labile organic carbon (LOC) contents with NT, CT treatments were obvious increased^25,26^. In the present study, our results indicated that SOC and LOC contents in paddy field were obvious changed under different tillage management’s conditions^15,16^. Compared with NT and RTO treatments, SOC and LOC contents at 0–10 cm and 10–20 cm layers in paddy field with combined application of tillage and crop residue incorporation practices (CT and RT) were increased considerably, which were possibly attributed to a larger proportion of organic compounds in crop residue^25^. On the other hand, higher decomposition rate of crop residue result in larger organic matter was the main reason for increase of SOC and LOC contents in paddy field with CT and RT treatments. This implied that a higher amount of organic material store through crop residues and tillage application were required for increasing the soil organic carbon (C) pool. Therefore, higher SOC and LOC contents in paddy field were obvious closely related to higher decomposition rate and organic matter content with crop residue input practices^27^. Meanwhile, our results showed that SOC and LOC contents in paddy field with NT treatment were increased marginally, compared to RTO treatment, these results were agree with the previous studies which the application of crop residue induce increase of SOC and LOC contents^26,28^, and mainly related to the initial soil C status, cropping ecosystem, quantity and quality of crop residue returning to field^2^.

In the present study, our results proved that SOC and LOC contents at 0–10 cm layer of CT, RT, NT and RTO treatments were larger than those of treatments at 10–20 cm layer, the main reason was attributed to that crop residue and fertilizer were incorporation into 0–10 cm soil layer in paddy field by the rotary tillage, but 10–20 cm soil layer was little disturbed under the applied with rotary tillage condition. Furthermore, crop residue and fertilizer provide abundant of soil available nutrient for rice root growth and soil microbial activities at 0–10 cm layer, and more C were added from crop residue return to paddy field. These results have been supported by many reports from other long-term field experiments^2,12^.

### Effects of different tillage managements on SOC mineralization characteristics

In this study, our results proved that soil enzyme activities (soil invertase, cellulose and urease) at 0–10 cm and 10–20 cm layers in paddy field with CT and RT treatments were significantly increased (Fig. 4). The reason may be explained by the higher contents of SOC and N in paddy field (Fig. 1)^16^, which were provided much C and N source substrate for soil invertase, cellulose and urease growth and multiply, and stimulate soil enzyme activities with crop residue input practices^29^. On the other hand, higher SOC content with CT and RT treatments could increase capacity of soil to protect extra-cellular pool of soil enzyme against proteolytic activity, and hence provided more functional enzyme molecules in paddy soil^30^, which suggested to provide higher C source for soil enzyme activities, and provide more soil nutrient and excellent soil ecological environment for soil enzyme multiplying and reduce competition for nutrient under CT and RT conditions^31^. In the present study, our results demonstrated that soil enzyme activities at 0–10 cm layer with all tillage treatments were higher than that of 10–20 cm layer, the main reason was attributed to that crop residue and fertilizer were incorporation into 0–10 cm layer in paddy field by the rotary tillage, which provide larger of soil available nutrient for rice root growth and soil microbial activities at 0–10 cm layer.

SOC mineralization was a vital biochemical process in paddy soil, it was mainly influenced through change of SOC content under different tillage management’s conditions. In this study, our results indicated that SOC mineralization was decreased rapidly at early stage of incubation time and gradually stabilized at the later stage of incubation time, which were consistent with previous results^6^. Compared to RTO treatment, SOC mineralization rate and SOC mineralization accumulation with CT, RT and NT treatments were increased, the main reason was attributed to that soil physical and chemical properties (eg., soil moisture and nutrient status were increased, soil compactness were decreased) were improved, especially the SOC content were also increased, which had an obvious influenced on mineralization process and SOC mineralization rate in paddy field. Meanwhile, this result demonstrated that SOC mineralization rate and accumulation at 0–10 cm layer with all tillage treatments were larger than that of 10–20 cm layer, the reason perhaps attributed to that input of organic matter (eg., crop roots and root exudates) were decreased with the increases of soil layer, which reduces the circulation and transformation of SOC^12^. The proportion of soil potential mineralization/SOC was the reflection of soil carbon fixation capacity in a certain extent^6^. In the present study, this result indicated that ratio of potential mineralization/SOC with combined application of tillage and crop residue incorporation managements were higher than that of without crop residue input management, the reason maybe attributed to that crop carbon fixation were increased, soil carbon emission were reduced, and SOC storage were also improved under crop residue incorporation conditions^2^.

Some results indicated that SOC content were provided main nutrient for soil microbial metabolism, and SOC mineralization were directly influenced by SOC content^2^. In this study, our results demonstrated that SOC mineralization and accumulation were significantly positively correlated with SOC content, soil invertase, cellulose, urease activities, but was significantly negatively correlated with soil pH, bulk density, suggested that SOC mineralization and accumulation were significantly influenced by SOC content, soil microorganisms and soil enzyme activities, which were agreed with previous results^10^. The reason may be attributed that input and output characteristics of SOC were changed through soil compactness under different tillage and crop residue incorporation conditions, and the retention capacity of SOC were increased with lower proportion of LOC content, higher SOC content were the main factor in promote SOC mineralization and accumulation^6^. On the other hand, it were provide more N and C sources for soil enzyme activities with tillage and crop residue incorporation practices, which can significantly increase the C in SOC that can be used by soil microorganism, then lead to influence on SOC mineralization and accumulation^32^. Meanwhile, this result demonstrated that soil mineralization constant was significantly negatively correlated with SOC content, soil invertase, cellulose activities, which were disagreed with previous result^33^. The reason maybe attributed to that beneficial to promote more stable soil aggregate structure and higher protection effect on soil organic carbon. Furthermore, soil mineralization constant were decreased with higher soil enzyme activities (invertase, cellulose and urease) in paddy field, suggested that soil water content the main factor in influence on SOC mineralization and enzyme activities under flooded irrigation condition. Therefore, these results suggested that soil mineralization constant were significantly influenced by soil physicochemical properties and nutrient (eg., ρb, SOC), soil microbial biomass carbon content, soil enzyme activities (invertase, cellulose and urease), which were closely related to different tillage managements.

## Conclusions

Our results clearly showed that 7 years of combined application of crop residues incorporation managements increased SOC and labile organic carbon contents compared to without crop residues input treatment in paddy field under the double-cropping rice system in southern of China. In the present study, our results demonstrated that SOC mineralization rate and accumulation at 0–10 cm and 10–20 cm layers in paddy field of CT, RT and NT treatments were higher than RTO treatment. Meanwhile, SOC mineralization rate and accumulation at 0–10 cm layer with CT, RT, NT and RTO treatments were larger than that of 10–20 cm layer. Compared with RTO treatment, it was benefit practices for improving SOC mineralization and soil carbon sequestration capacity in paddy field with NT and RT treatments. There was significantly positive correlation between SOC mineralization accumulation and SOC content, soil invertase, cellulose, urease activities, but it was significantly negatively correlated with soil pH, bulk density, respectively. In conclusion, combined application of tillage with crop residues incorporation managements were important strategy for improving SOC status and SOC mineralization in the double-cropping rice field in southern China.

## Data Availability

Data is provided within the manuscript.

## References

[1] Balesdent, J. *et al.* Atmosphere–soil carbon transfer as a function of soil depth. *Nature* **559**, 599–602 (2018). 29995858 10.1038/s41586-018-0328-3

[2] Li, S. *et al.* Dynamics of soil labile organic carbon fractions and C-cycle enzyme activities under straw mulch in Chengdu Plain. *Soil Till. Res.* **155**, 289–297 (2016). 10.1016/j.still.2015.07.019

[3] Zhao, F., Yang, G., Han, X., Feng, Y. & Ren, G. Stratification of carbon fractions and carbon management index in deep soil affected by the Grain-to-Green Program in China. *PLoS One* **9**, e99657 (2014). 24915425 10.1371/journal.pone.0099657 PMC4051785

[4] Plaza-Bonilla, D., Álvaro-Fuentes, J. & Cantero-Martínez, C. Identifying soil organic carbon fractions sensitive to agricultural management practices. *Soil Till. Res.* **139**, 19–22 (2014). 10.1016/j.still.2014.01.006

[5] Dimassi, B. *et al.* Effect of nutrients availability and long-term tillage on priming effect and soil C mineralization. *Soil Biol. Biochem.* **78**, 332–339 (2014). 10.1016/j.soilbio.2014.07.016

[6] Kan, Z. R. *et al.* Characteristics of carbon mineralization and accumulation under long-term conservation tillage. *Catena* **193**, 104636 (2020). 10.1016/j.catena.2020.104636

[7] Luo, Y. B., Wan, S. Q., Hui, D. F. & Wallace, L. L. Acclimatization of soil respiration to warming in a tall grass prairie. *Nature* **413**, 622–625 (2001). 11675783 10.1038/35098065

[8] Lal, R. Soil carbon sequestration impacts on global climate change and food security. *Science* **304**, 1623–1627 (2004). 15192216 10.1126/science.1097396

[9] Li, S. P. *et al.* Is least limiting water range a useful indicator of the impact of tillage management on maize yield?. *Soil Till. Res.* **199**, 104602 (2020). 10.1016/j.still.2020.104602

[10] Wang, H. H. *et al.* Long-term no-tillage and different residue amounts alter soil microbial community composition and increase the risk of maize root rot in northeast China. *Soil Till. Res.* **196**, 104452 (2020). 10.1016/j.still.2019.104452

[11] Niu, Y. H. *et al.* No-tillage did not increase organic carbon storage but stimulated N_2_O emissions in an intensively cultivated sandy loam soil: a negative climate effect. *Soil Till. Res.* **195**, 104419 (2019). 10.1016/j.still.2019.104419

[12] Sarker, J. R. *et al.* Tillage history and crop residue input enhanced native carbon mineralisation and nutrient supply in contrasting soils under long-term farming systems. *Soil Till. Res.* **193**, 71–84 (2019). 10.1016/j.still.2019.05.027

[13] Sauvadet, M. *et al.* High carbon use efficiency and low priming effect promote soil C stabilization under reduced tillage. *Soil Biol. Biochem.* **123**, 64–73 (2018). 10.1016/j.soilbio.2018.04.026

[14] Liu, C., Lu, M., Cui, J., Li, B. & Fang, C. M. Effects of straw carbon input on carbon dynamics in agricultural soils: A meta-analysis. *GCB Bioenergy* **20**, 1366–1381 (2014). 10.1111/gcb.12517 24395454

[15] Yang, X. Y., Ren, W. D., Sun, B. H. & Zhang, S. L. Effects of contrasting soil management regimes on total and labile soil organic carbon fractions in a loess soil in China. *Geoderma* **177–178**, 49–56 (2012). 10.1016/j.geoderma.2012.01.033

[16] Tang, H. M. *et al.* Effects of different soil tillage systems on soil carbon management index under double-cropping rice field in southern China. *Agron. J.* **111**, 440–446 (2019). 10.2134/agronj2018.06.0414

[17] Lee, K. S., Park, S. H., Park, W. Y. & Lee, C. S. Strip tillage characteristics of rotary tiller blades for use in a dryland direct rice seeder. *Soil Till. Res.* **71**, 25–32 (2003). 10.1016/S0167-1987(02)00159-9

[18] Ellert, B. H. & Bettany, J. R. Calculation of organic matter and nutrients stored in soils under contrasting management regimes. *Can. J. Soil Sci.* **75**, 529–538 (1995). 10.4141/cjss95-075

[19] Janzen, H. H., Campbell, C. A., Brandt, S. A., Lafond, G. P. & Townley-Smith, L. Light fraction organic matter in soils from long-term crop rotations. *Soil Sci. Soc. Am. J.* **56**, 1799–1806 (1992). 10.2136/sssaj1992.03615995005600060025x

[20] Blake, G.R. & Hartge, K.H. Bulk density. In: Klute, A. (Ed.), Methods of Soil Analysis. Part I: Physical and Mineralogical Methods Agronomy Monograph No. 9. ASA-SSSA, Madison, pp 363–375. (1986).

[21] Zhang, Q. *et al.* Fatty-acid profiles and enzyme activities in soil particle-size fractions under long-term fertilization. *Soil Sci. Soc. Am. J.* **80**, 97–111 (2016). 10.2136/sssaj2015.07.0255

[22] Bao, S. D. *Soil and Agricultural Chemistry Analysis* 49–56 (China Agriculture Press, 2000).

[23] Sinha, M. K., Sinha, D. P. & Sinha, H. Organic matter transformations in soils. V. Kinetics of carbon and nitrogen mineralization in soil amended with different organic materials. *Plant Soil* **46**, 579–590 (1977). 10.1007/BF00015917

[24] SAS. SAS Software of the SAS System for Windows. SAS Institute Inc, Cary, NC, USA. (2008).

[25] Liu, E. K. *et al.* Long-term effects of no-tillage management practice on soil organic carbon and its fractions in the northern China. *Geoderma* **213**, 379–384 (2014). 10.1016/j.geoderma.2013.08.021

[26] Guo, L. J., Zhang, Z. S., Wang, D. D., Li, C. F. & Cao, C. G. Effects of short-term conservation management practices on soil organic carbon fractions and microbial community composition under a rice-wheat rotation system. *Biol. Fertil. Soils* **51**, 65–75 (2015). 10.1007/s00374-014-0951-6

[27] Kalbitz, K. *et al.* The carbon count of 2000 years of rice cultivation. *Glob. Change Biol.* **19**, 1107–1113 (2013). 10.1111/gcb.12080 23504888

[28] Xu, M. *et al.* Soil organic carbon active fractions as early indicators for total carbon change under straw incorporation. *Biol. Fertil. Soils* **7**, 745–752 (2011). 10.1007/s00374-011-0579-8

[29] Pandey, D., Agrawal, M. & Bohra, J. S. Effects of conventional tillage and no tillage permutations on extracellular enzyme activities and microbial biomass under rice cultivation. *Soil Till. Res.* **136**, 51–60 (2014). 10.1016/j.still.2013.09.013

[30] Nannipieri, P., Sequi, P. & Fusi, P. Humus and enzyme activity. In *Humic Substances in Terrestrial Ecosystems* (ed. Piccolo, A.) 293–327 (Elsevier, 1996).

[31] Pandey, D., Agrawal, M. & Bohra, J. S. Effects of conventional tillage and no tillage permutations on extracellular soil enzyme activities and microbial biomass under rice cultivation. *Soil Till. Res.* **136**, 51–60 (2014). 10.1016/j.still.2013.09.013

[32] Wei, Z. Q., Wu, S. H., Zhou, S. L. & Chen, L. Installation of impervious surface in urban areas affects microbial biomass, activity (potential C mineralisation), and functional diversity of the fine earth. *Soil Res.* **51**, 59–67 (2013). 10.1071/SR12089

[33] Kan, Z. R. *et al.* Effects of experiment duration on carbon mineralization and accumulation under no-till. *Soil Till. Res.* **209**, 104939 (2021). 10.1016/j.still.2021.104939

